# Supplementary material for: Diabetes-Related Lower Extremity Amputations in Romania: Patterns and Changes between 2015 and 2019
Source: Int J Environ Res Public Health. 2022 Dec 29;20(1):557. doi: 10.3390/ijerph20010557 (PMC9819762; doi:10.3390/ijerph20010557)
Supplement: Supplementary file 1 [file ijerph-20-00557-s001.zip › Coman_supplementary.pdf]

Table S1. Absolute numbers of the different types of amputations recorded during the study years.

Amputation type (ICD 10 AM v.3 code)	2015	2016	2017	2018	2019
Major amputations					
Hip amputation (44370-00)					
Amputation above the knee (44367-00)					
Amputation below the knee (44367-02)					
Ankle amputation through the maleolli of the tibia and fibula (44361-01)					
Ankle disarticulation (44361-00)					
Minor amputations					
Midtarsal amputation (44364-00)					
Transmetatarsal amputation (44364-01)					
Toe amputation (44338-00)					
Toe amputation with metatarsal bone (44358-00)					
Toe disarticulation (90557-00)					

ICD 10, International Classification of Diseases 10th revision
